# A *Plasmodium falciparum* protein tyrosine phosphatase inhibitor identified from the ChEMBL‐NTD database blocks parasite growth

**DOI:** 10.1002/2211-5463.13171

**Published:** 2021-05-29

**Authors:** Rajan Pandey, Priya Gupta, Asif Mohmmed, Pawan Malhotra, Dinesh Gupta

**Affiliations:** ^1^ Translational Bioinformatics Group International Centre for Genetic Engineering and Biotechnology New Delhi India; ^2^ Malaria Biology Group International Centre for Genetic Engineering and Biotechnology New Delhi India; ^3^ Parasite Cell Biology Group International Centre for Genetic Engineering and Biotechnology New Delhi India

**Keywords:** enzymatic assay, inhibition assay, phosphatase, plasmodium, post‐translational modifications

## Abstract

Post‐translational modifications, especially reversible phosphorylation, are among the most common mechanisms that regulate protein function and biological processes in *Plasmodium* species. Of the *Plasmodium* phosphatases, phosphatase of regenerating liver (*Pf*PRL) is secreted and is an essential phosphatase. Here, we expressed *Pf*PRL in a heterologous expression system, and then purified and characterized its phosphatase activity. We found that Novartis_003209, a previously identified inhibitor, inhibited the *Pf*PRL phosphatase activity of recombinant *Pf*PRL and blocked *in vitro* parasite growth in a dose‐dependent manner. Further, *in silico* docking analysis of Novartis_003209 with all four *P. falciparum* tyrosine phosphatases (PTP) demonstrated that Novartis_003209 is a *Plasmodium* PTP inhibitor. Overall, our results identify a scaffold as a potential starting point to design a PTP‐specific inhibitor.

## Graphical abstract

*Plasmodium* PRL is a secreted protein tyrosine phosphatase and is essential for parasite survival. Our study shows that Novartis_003209 inhibits recombinant *Pf*PRL phosphatase activity and *in vitro* parasite growth in a dose‐dependent manner. Further, *in silico* docking studies with four *P. falciparum* tyrosine phosphatases suggest that the identified molecule could be a general plasmodium tyrosine phosphatase inhibitor.

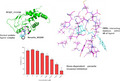

**Abbreviations**
aa: amino acid
HDP: heme detoxification protein
MCS: multiple cloning sites
OMF: 3‐*O*‐methylfluorescein
OMFP: OMF phosphate
PBS: phosphate‐buffered saline
*Pf*: *Plasmodium falciparum*
*Pf*YVH1: *Plasmodium falciparum* dual‐specificity protein phosphatase
PRL: phosphatase of regenerating liver
PTP: protein tyrosine phosphatase
RBC: red blood cell

Malaria, a parasitic disease caused by *Plasmodium*, is still one of the leading causes of death worldwide [1]. Although malaria cases have dropped significantly in the past decade, it is believed that they may rise again as a result of the emergence of resistance to current antimalarials [1, 2, 3]. Therefore, there is an urgent need to identify newer targets and develop a new pipeline of antimalarials. Reversible phosphorylation mediated by kinases and phosphatases is one of the most common mechanisms by which protein functions are regulated in eukaryotes and *Plasmodium* [4, 5, 6, 7, 8]. Phosphorylation balance is crucial for different parasites' developmental stages [5, 8]. Several genome‐wide and proteome‐wide studies have identified 100 kinases and 67 phosphatases in the *Plasmodium falciparum* genome, and many of these regulatory proteins are essential for the parasite life cycle [5, 8, 9, 10, 11, 12]. Therefore, these essential kinases/phosphatases are being considered important targets for the development of new antimalarials.

Protein tyrosine phosphatases (PTPs) are essential signaling enzymes that, together with protein tyrosine kinases, regulate diverse cellular processes such as cell motility, division, proliferation, and survival [13, 14, 15, 16]. As the name suggests, PTPs regulate tyrosine phosphorylation of proteins and have a distinct active site signature motif, HCX5R [15, 17]. Many human diseases, including cancer, diabetes/obesity, autoimmune disorders, and infectious diseases, have been linked to aberrant tyrosine phosphorylation [18, 19]. Therefore, many of these tyrosine phosphatases are being studied as therapeutic targets. For example, CDC25 has emerged as an important target for cancer and autoimmune diseases, PTP1b for obesity and type II diabetes and SHP‐2 for rheumatoid arthritis [16, 18, 20, 21]. We earlier illustrated the *P. falciparum* phosphatome and identified 67 phosphatases grouped into 13 superfamilies [10]. Thirty‐three of these phosphatases do not have any human homologs, and six of these phosphatases are *Plasmodium‐specific* phosphatases [10]. Among these phosphatases, phosphatases of regenerating liver (*Pf*PRL) family homolog is an essential PTP and colocalizes with AMA‐1, a membrane‐associated protein linked to red blood cell (RBC) invasion of *Plasmodium* merozoites [22]. We have previously explored *Pf*PRL as a drug target by *in silico* virtual screening of ChEMBL‐NTD library and identified a list of compounds. Among these compounds, Novartis_003209 was docked with *Pf*PRL with the least binding energy and showed stable interaction with *Pf*PRL [23].

In the present study, we cloned and expressed *Pf*PRL protein and established its phosphatase activity assay. Further, we analyzed the inhibitory potential of Novartis_003209 against the purified recombinant *Pf*PRL protein and performed comparative molecular docking analysis with the other *Plasmodium* PTPs catalytic site. The structure–activity relationship discussed in the present study may pave the way for the rational design of new inhibitor(s) targeting PTPs of the human malaria parasite *P. falciparum*.

## Methods

### Recombinant *Pf*PRL expression and protein purification

For *Pf*PRL PTP expression, the *Pf*PRL gene was amplified using Q5 polymerase, a forward primer (ATGAACTTGTGTCCA) with a *BamH*I site, and a reverse primer (CATAAAATGACATTT) incorporating *Sal*I site to express *Pf*PRL protein from amino acids 11N to 218 M. The PCR amplified product was inserted into the multiple cloning sites (MCS) sites of the pJET vector. Positive clones were sequenced, and the cloned fragment was inserted into the *BamHI*‐*SalI* sites of the pQE‐30 vector (6X‐HIS tag is located upstream of BamHI‐SalI (MCS) cloning sites). The positive clones for *Pf*PRL in the pQE‐30 vector were transformed in *Escherichia coli* m15 strain cells for protein expression. Recombinant N terminus HIS‐tagged‐*Pf*PRL was expressed for 8 h at 25 °C with 0.25 mm IPTG. Cells were harvested, and the cell pellet was resuspended in lysis buffer (50 mm Tris pH 7.5, 300 mm NaCl, 0.01% Triton, and 10% glycerol). Cells were lysed using lysis buffer by sonication; cell lysate was centrifuged at 14,300 *g* for 20 min. The supernatant solution containing the soluble protein was affinity‐purified to near purity using a Ni‐NTA^+^ column. The purity of the protein was analyzed on SDS/PAGE and by western blot analysis.

### Recombinant *Pf*PRL phosphatase activity assay

Phosphatase activity of nearly homogenous purified *Pf*PRL protein was analyzed using 3‐*O*‐methylfluorescein phosphate (OMFP) hydrolysis in a reaction mixture containing 50 mm sodium acetate (pH 5.5), 100 mm NaCl, 10 mm DTT, 20% glycerol, and (4 µm–1 mm) OMFP at 37 °C. The reaction was initiated by the addition of 4 µm of His‐tagged‐*Pf*PRL, and the release of 3‐*O*‐methylfluorescein (OMF) was monitored at 485/535 nm in Victor 1420 multilabel counter (Perkin Elmer, USA). His‐HDP (heme detoxification protein, a protein used in process of hemozoin formation [24, 25] and does not have phosphatase domain or show phosphatase activity) was used as a negative control. The compound Novartis_003209, identified from *in silico* study, was analyzed in an inhibition assay. The inhibitory potential of Novartis_003209 was tested against purified recombinant *Pf*PRL in the presence of 50 mm sodium acetate (pH 5.5), 100 mm NaCl, 10 mm DTT, 20% glycerol, and 50 µm OMFP at 37 °C. Novartis_003209 was procured from MolPort. Purity and other details for the received compounds may be retrieved from the MolProt site (https://www.molport.com/) with catalog numbers STK766939 (MolProt, Riga, Latvia). graphpad prism 9 (GraphPad Software, Inc, USA) software was used to plot phosphatase activity and inhibition assay graphs.

### Parasite growth inhibition assay

The effect of phosphatase inhibitors on parasite growth was evaluated on the 3D7 strains of *P. falciparum*. The parasite culture was synchronized using 5% sorbitol, and the assay was started at the schizont stage with hematocrit and parasitemia of synchronized schizont stage culture adjusted to 2% and 1%, respectively. Novartis_003209 (0.07–10 μm) was added to the parasite culture in separate 96‐well plates, and parasitemia was estimated after an incubation period of 24 h using flow cytometry. Briefly, cells from the samples were collected and washed with phosphate‐buffered saline (PBS), followed by staining with ethidium bromide (10 μg·mL^−1^) for 20 min at 37 °C in dark. The cells were subsequently washed twice with PBS and analyzed on FACSCalibur (Becton Dickinson, USA), using cellquest software (Becton Dickinson, USA). Fluorescence signal (FL2) was detected with the 590 nm band‐pass filter using an excitation laser of 488 nm, collecting 100 000 cells per sample. Uninfected RBCs stained in a similar manner were used as a control. Following data acquisition, each sample was analyzed for percentage parasitemia by determining the proportion of FL2‐positive cells using CellQuest.

### Comparative molecular docking of Novartis_003209 to other *Plasmodium* PTPs

NCBI BLASTp (https://blast.ncbi.nlm.nih.gov/Blast.cgi) and PlasmodDB (release 49) [26] were used to identify *P. falciparum* proteins showed similarity with *Pf*PRL. Three‐dimensional structure for none of 4 *Plasmodium* tyrosine phosphatases has been resolved yet, so homology and threading‐based techniques were used to generate a 3D structure for the *Plasmodium* PTPs [27, 28, 29]. The best‐predicted model was selected for molecular dynamic simulation for further optimization, validation, and downstream analysis. gromacs (ver 4.6.3) with the CHARMM27 force field was used to perform molecular dynamic simulation in an aqueous environment [30, 31]. The methods and parameters for the MD simulations were followed as mentioned in our previous study [23]. The final simulated protein structure's quality was verified using the Ramachandran plot [32]. Ligand preparation and docking were performed using raccoon and AutoDockTools4 [33, 34]. Autodock4 uses Lamarckian Genetic Algorithm and Empirical Binding Free Energy Function to calculate ligand's binding energy to the target protein [34]. pymol (https://pymol.org/2/) and LigPlus were used to visualize and generate images [35].

## Results

### *PfPRL* expression, purification, and its activity analysis

**Fig. 1:**
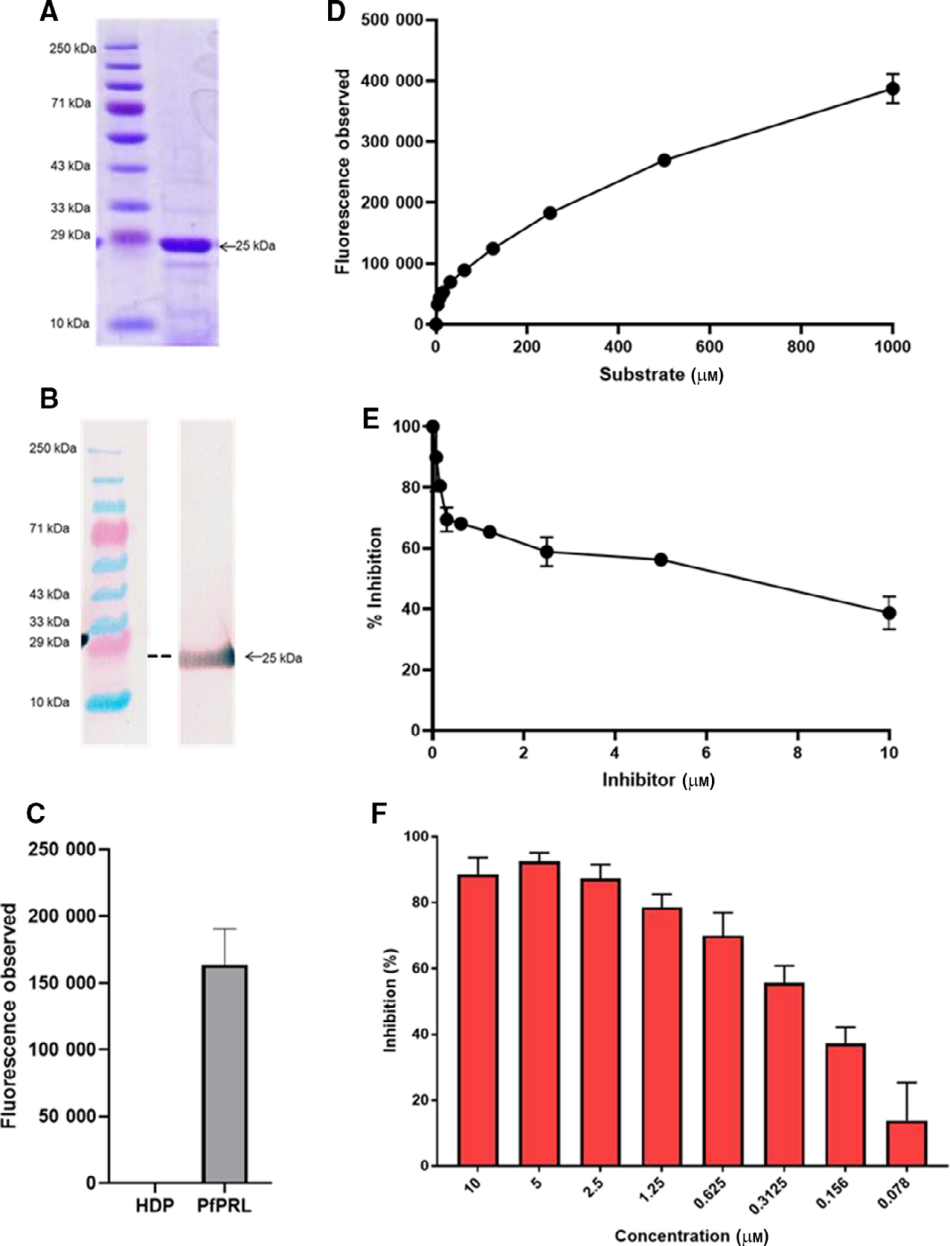
*Pf*PRL purification and activity analysis. (A) Coomassie‐stained gel of nearly purified *Pf*PRL protein using Ni‐NTA^+^ column (also see Fig. S7), (B) Western blot of *Pf*PRL using an anti‐HIS antibody and DAB (3,3'‐diaminobenzidine) substrate (intervening lane between molecular marker and *Pf*PRL has been removed, see Fig. S7). (C) The figure shows the hydrolysis of the substrate (200 μm) with *Pf*HDP (4 μm) and *Pf*PRL (4 μm) in the presence of 50 mm sodium acetate, pH5.5, 100 mm NaCl, 10 mm DTT, and 20% glycerol. (D) The figure represents hydrolysis of OMFP substrate by *Pf*PRL, measured in a reaction mixture containing 50 mm sodium acetate, pH5.5, 100 mm NaCl, 10 mm DTT, 20% glycerol, 4 μm HIS‐*Pf*PRL and varying concentrations of OMFP. (E) Figure represents hydrolysis of OMFP by recombinant *Pf*PRL, measured in the presence of different concentrations (0–10 μm) of inhibitor Novartis_003209. Fluorescence measurements at 485/535 nm were obtained following 90‐min incubation at 37 °C. The data represent the mean ± SD (standard deviation) of three independent experiments. (F) Dose‐dependent parasite growth inhibition during asexual blood stages using *Pf*PRL phosphatase identified inhibitor, Novartis_003209, based on *in silico* drug screening and recombinant *Pf*PRL inhibition assay results (number of independent experiment (n)=3).

We have earlier reported an *in silico* drug screening analysis against *Pf*PRL and identified nine potential hits. Among these hits, Novartis_003209 showed the lowest free binding energy [23]. To validate the inhibitory activity of Novartis_003209, we cloned and expressed *Pf*PRL protein (amino acid 11N–218 M) in an *E. coli* heterologous expression system (Fig. 1A,B). SDS/PAGE and western blot analysis using anti‐HIS antibody showed expression of recombinant *PfPRL* with the apparent size of ~ 25 kDa (Fig. 1A,B). Solubility studies revealed that recombinant protein was expressed in both soluble and pellet fractions in *E. coli* expression system (Fig. 1C). We purified the recombinant *PfPRL* protein on the Ni‐NTA^+^ column. And the purified protein was analyzed on SDS/PAGE and western blot using an anti‐His antibody. As shown in Fig. 1A, PfPRL was purified with more than 85 % purity.

To functionally characterize the recombinant *Pf*PRL protein, we performed the phosphatase activity analysis using OMFP as a substrate in a reaction mixture containing 50mM sodium acetate (pH 5.5), 100 mm NaCl, 10 mm DTT, 20% glycerol and (4 µm–1 mm) OMFP at 37 °C as described earlier [22]. Recombinant *Pf*PRL hydrolyzed OMFP in a dose‐dependent manner (Fig. 1C,D). Next, we tested the inhibitor's effect, Novartis_003209, which showed the highest in *silico* binding affinity with the *Pf*PRL on OMFP hydrolysis mediated by *PfPRL*. Novartis_003209 showed a dose‐dependent inhibition of OMFP hydrolysis with 50% inhibition at a value of < 10 µm (IC_50_ value, 5.175 µm, 95% Cl profile likelihood 3.515–8.705 µm, Fig. 1E). Together, the results showed that Novartis_003209 binds to *Pf*PRL and inhibits its phosphatase activity significantly.

### The presence of Novartis_003209 blocks parasite growth during blood stages of *P. falciparum* life cycle

Phosphatase of regenerating liver has been shown to play a crucial role in parasite growth and survival as the attempts to generate its knockout were unsuccessful [5, 36]. Further, *Pf*PRL has been shown to be an extracellular secreted protein at asexual blood stages of *P. falciparum* [37]. Therefore, to investigate the role of *Pf*PRL at asexual blood stages, we tested the effect of Novartis_003209 on parasite growth in human RBCs in an *in vitro P. falciparum* culture. The mature schizont stage parasites (3D7 strain) at 2% hematocrit and 1% parasitemia were treated with the phosphatase inhibitor Novartis_003209 (0.07–10 μm) in a 96‐well cell culture plate. The parasitemia was estimated 24 h postinfection in control and treated samples. A dose‐dependent decrease in the parasitemia was observed in Novartis_003209‐treated samples with a maximum of ~ 90% reduced parasitemia at 5 µm concentration with the IC_50_ value of 0.273 µm (95% Cl profile likelihood, 0.2314 to 0.3189 µm; Fig. 1F), indicating a potential role of *Pf*PRL in parasite growth during blood stages. Interestingly, growth inhibition assay results showed that Novartis_003209 blocked 50% parasite growth at a significantly lower concentration than what it showed for blocking the *in vitro* recombinant phosphatase activity. Thus, these results advocated that Novartis_003209 may have other potential targets, including *Pf*PRL phosphatase.

### *In* *silico* docking revealed Novartis_003209 to have an affinity for the PTP catalytic site

**Fig. 2:**
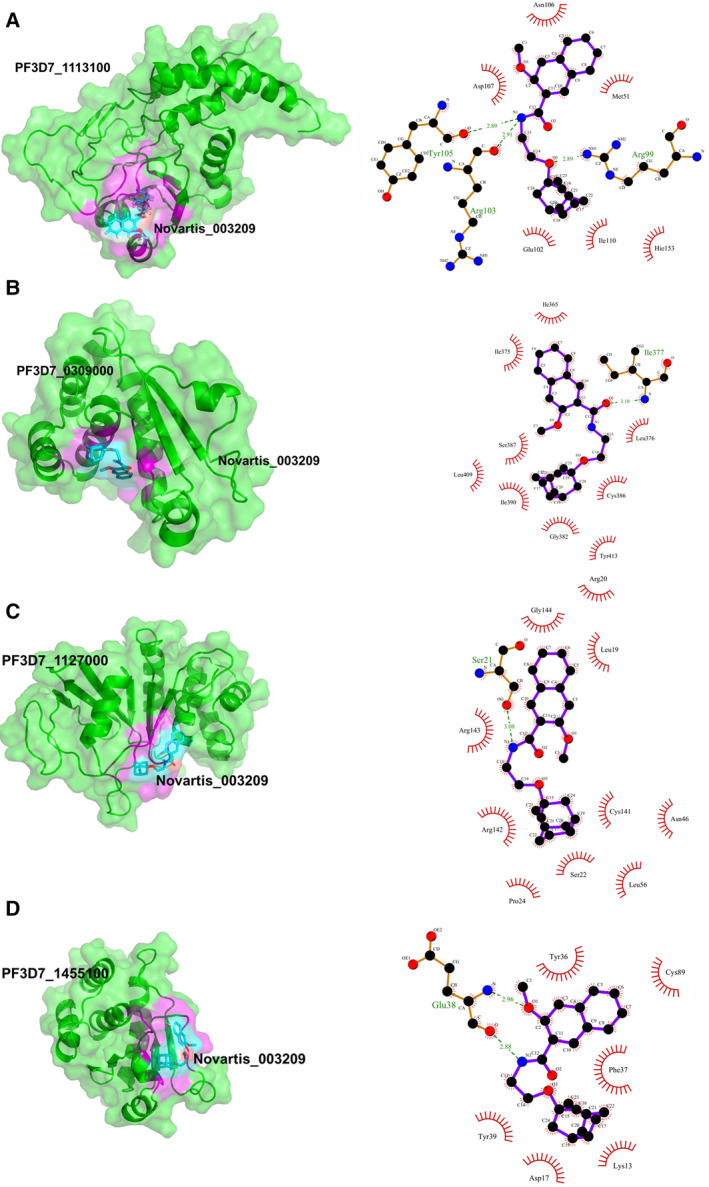
Molecular docking of Plasmodium PTPs; PfPRL (A), PF3D7_0309000 (B), PF3D7_1127000 (C), and PF3D7_1455100 (D) with Novartis_003209. Left ‐ Figure represents a cartoon and surface view of the protein‐ligand complex. Interacting PTP residues within 4 Å of the ligand (cyan) have been shown in magenta color. Right – Ligplot image showing interacting PTP residues.

As the Novartis_003209 showed a lower IC_50_ value in an *in vitro* parasite growth assay than its effect on recombinant *Pf*PRL activity, we speculated that Novartis_003209 might be targeting other *P. falciparum* proteins. Therefore, we searched for any *P. falciparum* proteins with a minimum of 30% sequence coverage and ≥ 30% sequence identity with *Pf*PRL. In addition to this, we also included PTPs encoded by *P. falciparum*, as PTPs share a conserved catalytic domain, and Novartis_003209 may be targeting the conserved PTP domain. The BLASTp search with *Pf*PRL sequence did not show any similar *P. falciparum* proteins. The three other PTPs with conserved HCX5R motif: PF3D7_1127000, PF3D7_0309000, and PF3D7_1455100, showed domain similarity with *Pf*PRL. To validate the hypothesis that Novartis_003209 may be targeting the other three PTPs, we performed multiple sequence alignment (MSA) of the four *P. falciparum* PTPs which showed the presence of conserved HCX5R motif (Fig. S2). The MSA analysis revealed that histidine (H) is replaced with cysteine (C) in the signature PTP motif region of PF3D7_1455100 (Fig. S2). As no crystal structure was available and attempts to generate full homology‐based models failed, we generated partial 3D coordinates of PF3D7_0309000 domain (312‐432 amino acid (aa); PDB template ID: 4KI9) and PF3D7_1455100 domain (2‐143; PDB template ID: 1ZZW), using SWISSMODEL. A single template did not predict 3D coordinates for the complete PF3D7_1127000 PTP domain (122‐276 aa); hence, a 3D model was generated using the Phyre2 server. Molecular dynamic simulation of the three predicted PTPs showed stable protein conformation, further validated by Ramachandran plot analysis (Figs S3–S5). Next, molecular docking analysis was performed in triplicate using AutoDockTools to check the binding affinity of Novartis_003209 with the 3 PTPs. The comparative binding energy analysis revealed the affinity of Novartis_003209 for the 3 PTPs, highest for the PF3D7_0309000 (binding energy: ~ 7.7 kcal/mol) but comparatively lower than *Pf*PRL (difference of 1.5 kcal/mol) and lowest for the PF3D7_1127000 (binding energy: ~ 6.45 kcal/mol; Table 1). The molecular docking analysis showed the binding of Novartis_003209 in the catalytic pocket for all the four PTPs, involving at least one residue from the conserved catalytic PTP motif HCX5R within 4 Å (HIS153 for *Pf*PRL, CYS379 for PF3D7_0309000, CYS141 for PF3D7_1127000, and CYS89 for PF3D7_1455100). These observations show that Novartis_003209 potentially targets other *Plasmodium* PTPs *in vivo* and the binding is PTP specific rather than *Pf*PRL (Figs 2 and S6). Comparative molecular docking studies of Novartis_003209 with human PRL‐3 (PDB ID: 1V3A) showed binding of −6.01 kcal/mol, showing Novartis_003209 has a higher affinity for *Pf*PRL compared with human phosphatase of regenerating liver (PRL) protein [23]. Further, previous bioassay studies, including cytotoxicity against human hepatocellular carcinoma cell line (Huh7), show that Novartis_003209 is either inactive or acts at > 100 µm concentration, suggesting that Novartis_003209 acts on human proteins and cells at a comparatively very high concentration as compared to the human malaria parasite (https://pubchem.ncbi.nlm.nih.gov/compound/2928525). Together, the study has identified Novartis_003209 as an inhibitor that binds to *Pf*PRL and other *Plasmodium* tyrosine phosphatases to inhibit the phosphatase activity and block the parasite growth.

**Table 1:** Autodock molecular docking binding energy score (kcal/mol) for *Plasmodium falciparum* PTPs.

Plasmodium PTPs	Replicate 1	Replicate 2	Replicate 3
PF3D7_1113100 (*Pf*PRL)	−9.27^a^		
PF3D7_0309000 (*Pf*YVH1)	−7.7	−7.73	−7.62
PF3D7_1127000	−6.6	−6.47	−6.3
PF3D7_1455100	−6.8	−6.77	−6.71

^a^ From previous *in silico* screening [23].

## Discussion

Reversible phosphorylation is a fundamental regulatory cellular process that governs several cellular functions required for cell survival, such as growth, division, adhesion, and motility. This process is mediated by kinases and phosphatases, to which a delicate balance is required for the successful completion of these cellular processes. Dysregulation of protein phosphorylation or alterations in phosphorylation patterns is a primary cause of many human diseases such as cancers, diabetes, autoimmune disorders, and neurological disorders [13, 14, 18, 19, 20]. Kinases and phosphatases are fast emerging as novel drugable targets for many diseases, including diseases caused by various pathogens such as *Plasmodium* [13, 14, 18, 19, 20, 21, 38]. We have carried out genome‐wise phosphatome analysis of *P*. *falciparum* genome previously and identified four tyrosine phosphatases in the genome [10]. Subsequently, we targeted one of the *Plasmodium* tyrosine phosphatases, *Pf*PRL, a secretory and an essential phosphatase for *in silico* inhibitor screening using the ChemBL‐NTB database [23]. Here, we characterize one of the inhibitors from the ChemBL‐NTB database, Novartis_003209, for its potential inhibitor activity as well as for the antiparasitic effects.

A previous study has expressed *Pf*PRL as a fusion protein with GST and showed that the recombinant *Pf*PRL functions possess phosphatase activity and are localized in the same compartment as *Pf*AMA‐1, suggesting its role in merozoite invasion or egress [22]. In the present study, we expressed the *Pf*PRL gene in an *E. coli* expression vector, pQE‐30, that adds a small tag 6X‐HIS at the N‐terminal of the *Pf*PRL protein. Next, we characterized the phosphatase activity of the recombinant *Pf*PRL protein by measuring the hydrolysis of OMFP and analyzed the effect of Novartis_003209 on the hydrolysis of OMFP as Novartis_003209 had been earlier shown to bind *Pf*PRL by *in silico* docking studies. Novartis_003209 inhibited the phosphatase activity of *Pf*PRL in a dose‐dependent manner with an IC_50_ value of < 10 µm. These results are in line with a previous report that has shown a block in OMFP inhibition by *Pf*PRL in the presence of various general phosphatase inhibitors such as sodium orthovanadate and phosphate inhibitor set [22]. Next, we measured the effect of Novartis_003209 in an *in vitro* parasite growth inhibition assay by adding different concentrations of inhibitor at the mature schizont stage of the *P*. *falciparum* parasites. A dose‐dependent decrease in parasitemia was observed in the Novartis_003209‐treated samples, with an IC_50_ of 0.273 µm.

The difference in IC_50_ values observed for Novartis_003209 inhibition for the enzyme activity vs. on the parasite growth prompted us to check whether Novartis_003209 may be acting on other *Plasmodium* proteins, especially other *Plasmodium* tyrosine phosphatases or observed phenotype is solely due to changes in the *Pf*PRL phosphatase activity as *Pf*PRL is an essential phosphatase for parasite survival and small changes in its activity may substantially dysregulate critical parasite processes. To validate the hypothesis that Novartis_003209 may be binding to other parasite proteins, we searched for *P. falciparum* proteins showing similarity with *Pf*PRL. Interestingly, none of the *Plasmodium* proteins showed similarity > 30% to *Pf*PRL. Next, we performed comparative protein‐ligand docking studies for Novartis_003209 with all the four *Plasmodium* PTPs: PF3D7_1113100 (*Pf*PRL, from previous study [23]), PF3D7_0309000 Plasmodium falciparum dual‐specificity protein phosphatase (*Pf*YVH1), PF3D7_1127000, and PF3D7_1455100 and compared these binding affinities with the human PRL. Among these PTPs, *Pf*PRL and *Pf*YVH1 have been functionally characterized and deemed essential for the parasite survival and growth [5, 22, 39]. Although not much work has been performed regarding the other two Plasmodium PTPs, however, genome‐wide knockout studies have shown that the two other PTPs are redundant. The comparative docking studies showed Novartis_003209 binds the catalytic sites of all the four *Plasmodium* PTPs, with PF3D7_0309000 showing binding energy comparable to *Pf*PRL. Together, the data advocate that Novartis_003209 is a *Plasmodium* tyrosine phosphatase‐specific inhibitor. This also explains the lower IC_50_ for *in vitro* parasite culture and targets all the *Plasmodium* PTPs. Further, Novartis_003209 is inactive on human cells and proteins (https://pubchem.ncbi.nlm.nih.gov/compound/2928525), suggesting that Novartis_3209 could be further explored as a potential antimalarial [23].

In summary, present study supports our previous *in silico* findings, where we identified nine potential inhibitors targeting *P. falciparum* tyrosine phosphatase, *Pf*PRL. Here, we show that Novartis_003209, inhibitor with the lowest free binding energy against *Pf*PRL in the *in silico* screening, blocks *Pf*PRL phosphatase activity and inhibits parasite growth. Additionally, the molecular docking simulations showed the binding affinity of Novartis_003209 to all the *Plasmodium* PTPs, suggesting that it could be a general *Plasmodium* tyrosine phosphatase inhibitor. It will be interesting to develop diverse compounds based on the structural scaffold of Novartis_003209 to make it target specific and study the efficacy of diverse yet related molecules to develop new and efficacious antimalarial.

## Conflict of interest

The authors declare no conflict of interest.

## Author contributions

PM and DG conceived the idea. RP performed *in silico* molecular docking and molecular dynamic studies. PG and RP performed *in vitro* experiments. RP, PG, PM, and DG performed the analysis. AM, PM, and DG supervised the study. RP, DG, and PM wrote the manuscript and all authors contributed to the manuscript.

## Supporting information


**Fig. S1.** Recombinant *Pf*PRL expression. (A) PCR amplified *Pf*PRL using Q5 polymerase, (B) *BamHI* and *SalI* digested PfPRL clone in PJET, (C) Subcellular localization of recombinant *Pf*PRL protein in *E. coli* expression system.
**Fig. S2.** Multiple sequence alignment of four *Plasmodium* PTPs with conserved HCX5R motif.
**Fig. S3.** Molecular dynamics simulation showing stable conformations of the predicted 3D structures of PF3D7_0309000. (A) Number of energy minimization steps required to achieve maximum force less than 1000 kJ mol‐1 nm‐1. (B) Fluctuations in temperature at constant volume (isothermal‐isochoric process) show that the system reaches the target temperature (300K) quickly and remain stable over the remainder of the equilibration. (C–D) Fluctuations in pressure and density at a constant temperature. (E–F) Root mean square deviation (RMSD) calculation using protein backbone structure and radius of gyration fluctuations during the 10 ns production simulation. Each analysis shows fluctuations within 2Å, suggesting correct and stable protein fold prediction for PF3D7_0309000. Additionally, during 10ns molecular dynamics run, the protein structure did not break, confirming stable predicted 3D structure. (G) Ramachandran plot analysis to check the quality of 3D model.
**Fig. S4.** Molecular dynamics simulation showing stable conformations of the predicted 3D structures of PF3D7_1127000. (A) Number of energy minimization steps required to achieve maximum force less than 1000 kJ mol‐1 nm‐1. (B) Fluctuations in temperature at constant volume (isothermal‐isochoric process) show that the system reaches the target temperature (300K) quickly and remain stable over the remainder of the equilibration. (C–D) Fluctuations in pressure and density at a constant temperature. (E–F) Root mean square deviation (RMSD) calculation using protein backbone structure and radius of gyration fluctuations during the 10 ns production simulation. Each analysis shows fluctuations within 2Å, suggesting correct and stable protein fold prediction for PF3D7_1127000. Additionally, during 10ns molecular dynamics run, the protein structure did not break, confirming stable predicted 3D structure. (G) Ramachandran plot analysis to check the quality of 3D model.
**Fig. S5.** Molecular dynamics simulation showing stable conformations of the predicted 3D structures of PF3D7_1455100. (A) Number of energy minimization steps required to achieve maximum force less than 1000 kJ mol‐1 nm‐1. (B) Fluctuations in temperature at constant volume (isothermal‐isochoric process) show that the system reaches the target temperature (300K) quickly and remain stable over the remainder of the equilibration. (C–D) Fluctuations in pressure and density at a constant temperature. (E–F) Root mean square deviation (RMSD) calculation using protein backbone structure and radius of gyration fluctuations during the 10 ns production simulation. Each analysis shows fluctuations within 2Å, suggesting correct and stable protein fold prediction for PF3D7_1455100. Additionally, during 10ns molecular dynamics run, the protein structure did not break, confirming stable predicted 3D structure. (G) Ramachandran plot analysis to check the quality of 3D model.
**Fig. S6.** Molecular docking of *Plasmodium* PTPs with Novartis_003209. (A, C, E and G) show the interacting residues of *Pf*PRL, PF3D7_0309000, PF3D7_1127000 and PF3D7_1455100 within 4 Å of Novartis_003209, respectively in the cartoon view. (B, D, F and H) display the interacting residues of *Pf*PRL, PF3D7_0309000, PF3D7_1127000 and PF3D7_1455100 within 4 Å of Novartis_003209, in the lines view and the hydrogen bond between protein‐ligand complex, using PyMol.
**Fig. S7.** Raw images of Fig. 1A,B.

## Data Availability

Predicted 3D models have been deposited in the Protein Model Database (PMDB) under the accession PM0084090, PM0084091, PM0084092, and PM0084093 for PF3D7_1113100, PF3D7_0309000, PF3D7_1455100, and PF3D7_1127000, respectively. Additional data will be available from the corresponding authors upon reasonable request.
