# Supplementary material for: A Plasmodium falciparum protein tyrosine phosphatase inhibitor identified from the ChEMBL‐NTD database blocks parasite growth
Source: FEBS Open Bio. 2021 May 29;11(7):1921–9. doi: 10.1002/2211-5463.13171 (PMC8255846; doi:10.1002/2211-5463.13171)

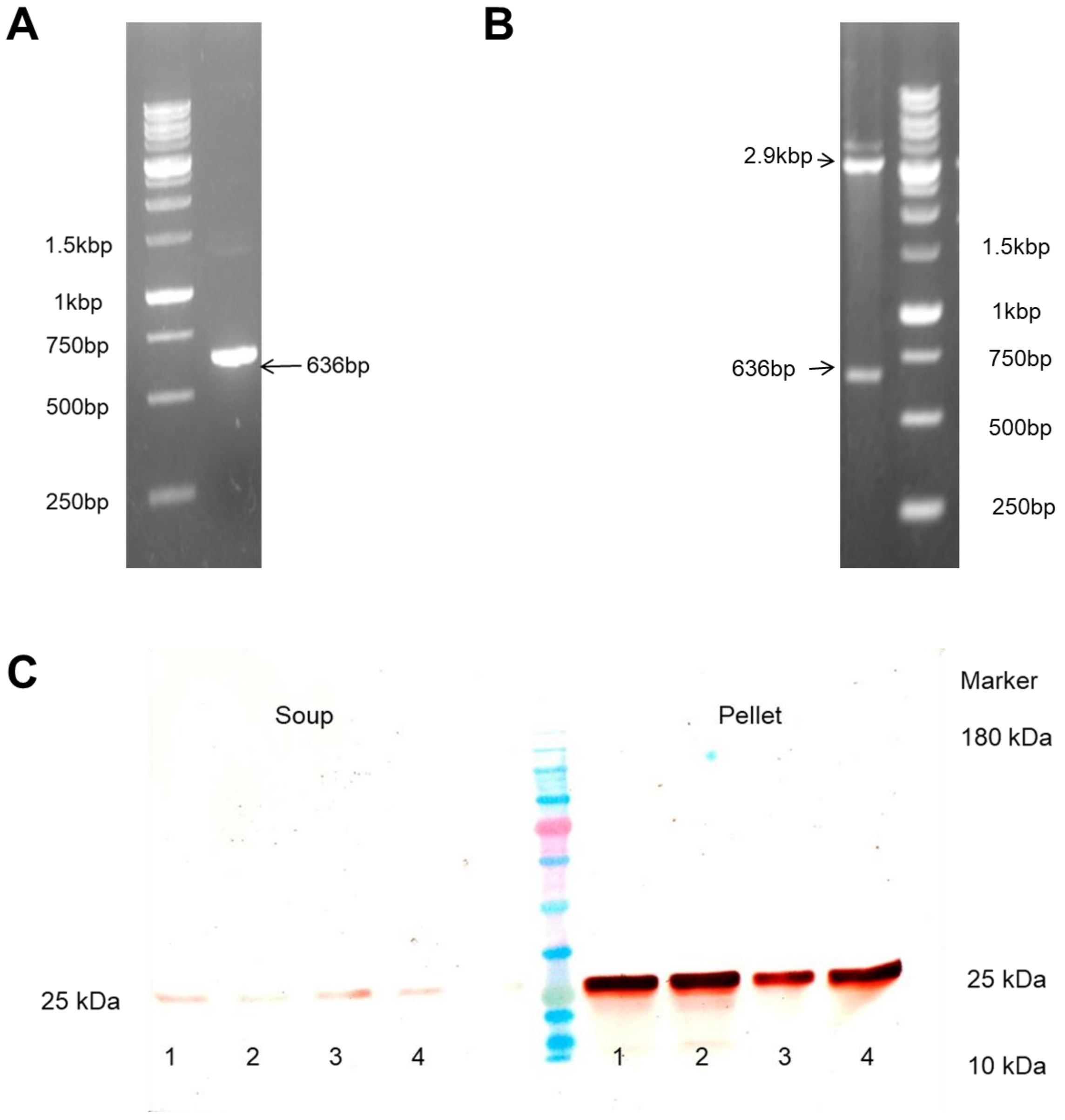
A
1.5kbp
1kbp
750bp
636bp
500bp
250bp
B
2.9kbp
636bp
1.5kbp
1kbp
750bp
500bp
250bp
C
Soup
Pellet
Marker
180 kDa
25 kDa
25 kDa
10 kDa
1
2
3
4
1
2
3
4

```
PF3D7_1113100    ------------------------------------------------------------
PF3D7_1127000    ------------------------------------------------------------
PF3D7_0309000    MIVKVFDYIYISNVYNANDIYELIKLNIGGVLTCFDCTCIEWCHHNDTNVTNKIFYKDIF
PF3D7_1455100    ------------------------------------------------------------

PF3D7_1113100    ------------------------------------------------------------
PF3D7_1127000    ---------------------------------------------MWNKLNDAENTDVDN
PF3D7_0309000    VNTKKDLIKCDVPIITNKSVNSDIIGGTHQINNYYNEQNNNYHDNTYKEFTQTHKTNIDP
PF3D7_1455100    ------------------------------------------------------------

PF3D7_1113100    -----------------------------------------MKSLENNEMHNLCPIHYYF
PF3D7_1127000    HGEPRNGFFQRLKKYKTFFYLFFGFL---------------MASLIIY-----AFYEYYF
PF3D7_0309000    SQIKSDHINE--ERKEHYDYIIFPSDIINNTQCNNNNLKDYIKSMLILK--EDAYIDFDV
PF3D7_1455100    ------------------------------------------------------------

PF3D7_1113100    NGRDYNSDTIIN--NVNIKYFNMDNCLGNANLHMDYLNPVLNHPTK--IEH---------
PF3D7_1127000    --------KK--------NGDFKLNCNDLNARCLNYKFPSFKPERLHIVDV---NTEN-N
PF3D7_0309000    IHMDQLKNKHNNNNNNNNNNNNNNNNNNNNNNCCTFKNPDISNTSQHHVEHIQIHKSNSH
PF3D7_1455100    ------------------------------------------------------------

PF3D7_1113100    -----G---------------KIKILILDAPTNDLLPLYIKEM-----------------
PF3D7_1127000    NYILRSSIPLFNGVYSEE---------------KLL-SYIKKLFEENNLSYSDNLTLHII
PF3D7_0309000    SNIPSDNINFCNKKYDKNLSRSVEISEKDKHPENSL-LY--EFVNKDKLNYKINQEEDTV
PF3D7_1455100    -------------------------------MIQII-PY--LYLGKKN-------DIDNV
                                                  . :  *

PF3D7_1113100    ------------------------------KNYNVTDLVRTCERT-YNDGEIQDAGINVH
PF3D7_1127000    SFLRNDLKEGCSYTSEHCFNKKNNI-------FNHII-----VGHQENPYDINED---EI
PF3D7_0309000    SSE----------KNKLCDNNNNNNMVHTRHIYNVCELNKCLRENKLIPYNNIYK---MK
PF3D7_1455100    EN--------------L----KKN---------NIKAVVICCTYFEYPEYKIPNG---YE
                                                  *                .
                                                          HCX5R
PF3D7_1113100    ELIFPDGDAPTEDIVSN---WLNIVNNVIKNNCAVAVHCVAGLGRAPVLASIVLIEFGMD
PF3D7_1127000    DDKLKSMSWNTDNLINQIKDL--KQKFNTMKNTIFFIHCRRGRDRTGEFVSAYKMIEQNK
PF3D7_0309000    HLYLNILDTFDENILKHVNKAHLFIDSVIQKKKNILIHCMAGISRCSSIILSYVSKKNKK
PF3D7_1455100    ILRINLEDIGLENISSYFEESNNFIHSYITKEQSVLICCCHGISRSSTISIAYLIGKQNF
                    :   .   ::: .         .    ::  . : *  * .*   :

PF3D7_1113100    PIDAI-VFIRDRRKGAINKRQ---LQFLKEYRKKKKKKNCLRKCHFM-------------
PF3D7_1127000    DFNSIVEENE--EIGKVKQQYVNMQKWLCLYLERIMKNPNVKCFNFL-------------
PF3D7_0309000    GIEYNFNLLK--SKYPFAHPNENFYRQLLLYEKMNYTLDG--CTDYHNIYKKIKMNRENL
PF3D7_1455100    GLNEAFNFIM--GKKNI-CPNIGFMEQLCEYEKRLKNQITFSSVKYISW-----------
                  ::             .        . *  * :   .       .:

PF3D7_1113100    ------------------------------------------------------------
PF3D7_1127000    ------------------------------------------------------------
PF3D7_0309000    EELKILNLKNDKQPIYNFRCKHCNYVLFNDNEIIKHDFKISKIKKNYGNSCTSIFIEKKE
PF3D7_1455100    -----------------FTSERCDKN-------VSTDFSLs-------------------

PF3D7_1113100    -----------------------------------------------------------
PF3D7_1127000    -----------------------------------------------------------
PF3D7_0309000    WILTENKMKGVLNCPNVNCNIKLGKWSWTGICCSCGYLQIPAFMINSSNVDRMNISKTV
PF3D7_1455100    -----------------------------------------------------------
```

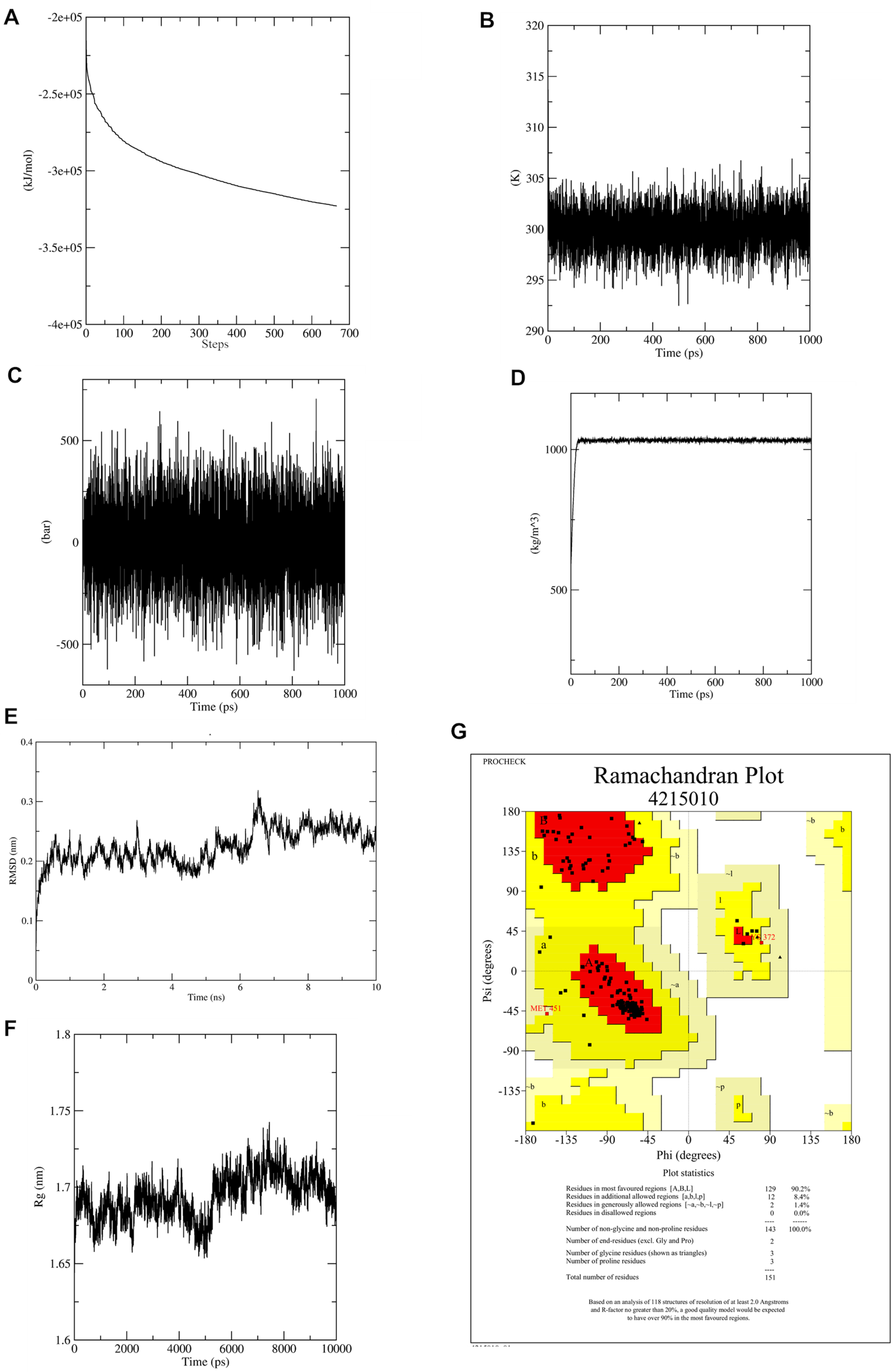
A
(kJ/mol)
Steps
B
(K)
Time (ps)
C
(bar)
Time (ps)
D
(kg/m^3)
Time (ps)
E
RMSD (nm)
Time (ns)
F
Rg (nm)
Time (ps)
G
PROCHECK
Ramachandran Plot
4215010
Psi (degrees)
Phi (degrees)
MET 451
Plot statistics
Residues in most favoured regions [A,B,L] 129 90.2%
Residues in additional allowed regions [a,b,l,p] 12 8.4%
Residues in generously allowed regions [~a,~b,~l,~p] 2 1.4%
Residues in disallowed regions 0 0.0%
Number of non-glycine and non-proline residues 143 100.0%
Number of end-residues (excl. Gly and Pro) 2
Number of glycine residues (shown as triangles) 3
Number of proline residues 3
Total number of residues 151
Based on an analysis of 118 structures of resolution of at least 2.0 Angstroms
and R-factor no greater than 20%, a good quality model would be expected
to have over 90% in the most favoured regions.

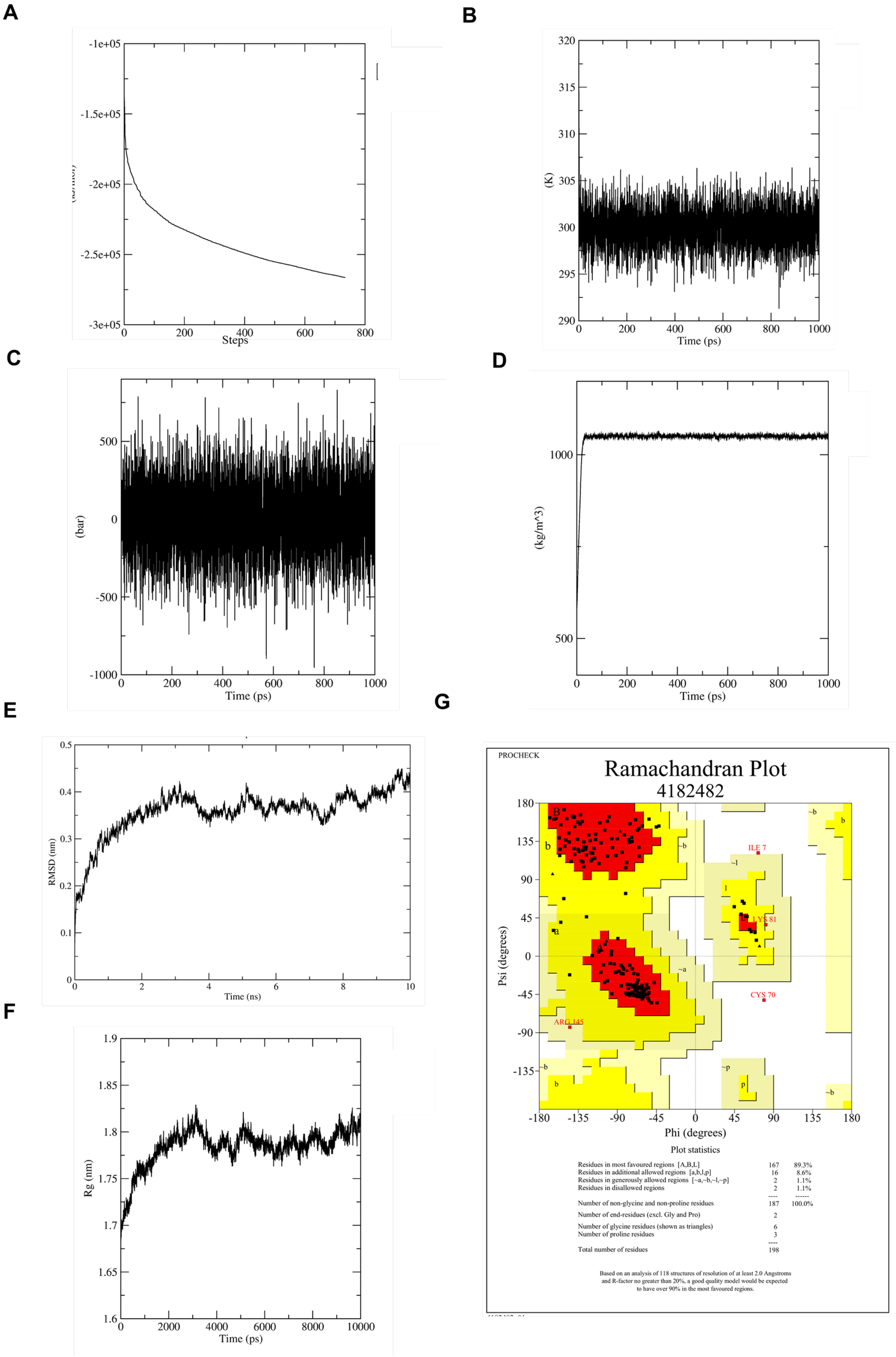

A
-1e+05
-1.5e+05
-2e+05
-2.5e+05
-3e+05
Steps
B
(K)
Time (ps)
C
(bar)
Time (ps)
D
(kg/m^3)
Time (ps)
E
RMSD (nm)
Time (ns)
F
Rg (nm)
Time (ps)
G
PROCHECK
Ramachandran Plot
4182482
Psi (degrees)
Phi (degrees)
ILE 7
LYS 81
CYS 70
ARG 145
Plot statistics
Residues in most favoured regions [A,B,L] 167 89.3%
Residues in additional allowed regions [a,b,l,p] 16 8.6%
Residues in generously allowed regions [~a,~b,~l,~p] 2 1.1%
Residues in disallowed regions 2 1.1%
Number of non-glycine and non-proline residues 187 100.0%
Number of end-residues (excl. Gly and Pro) 2
Number of glycine residues (shown as triangles) 6
Number of proline residues 3
Total number of residues 198
Based on an analysis of 118 structures of resolution of at least 2.0 Angstroms and R-factor no greater than 20%, a good quality model would be expected to have over 90% in the most favoured regions.

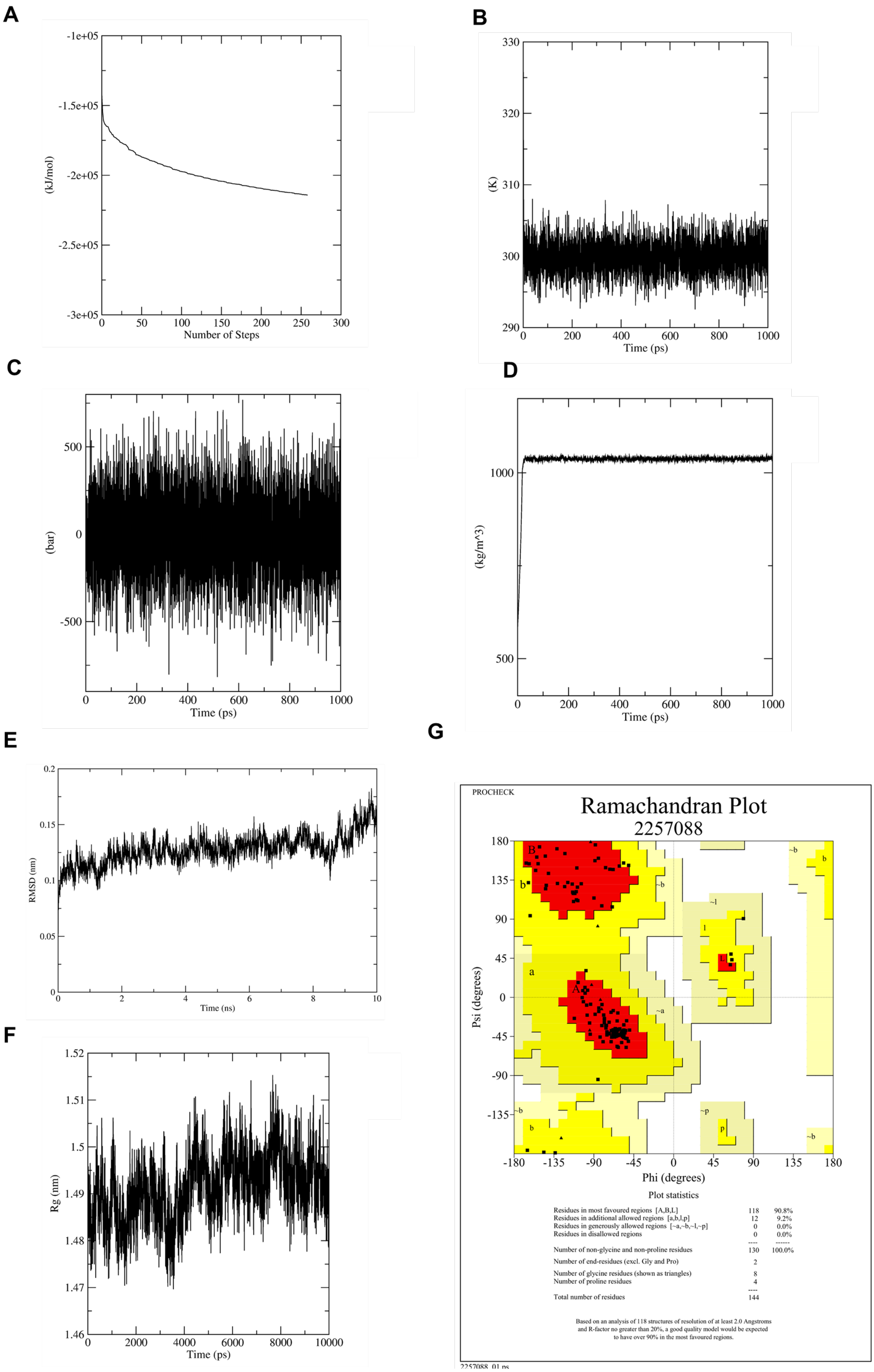
A
(kJ/mol)
Number of Steps
B
(K)
Time (ps)
C
(bar)
Time (ps)
D
(kg/m^3)
Time (ps)
E
RMSD (nm)
Time (ns)
F
Rg (nm)
Time (ps)
G
PROCHECK
Ramachandran Plot
2257088
Psi (degrees)
Phi (degrees)
Plot statistics
Residues in most favoured regions [A,B,L] 118 90.8%
Residues in additional allowed regions [a,b,l,p] 12 9.2%
Residues in generously allowed regions [~a,~b,~l,~p] 0 0.0%
Residues in disallowed regions 0 0.0%
Number of non-glycine and non-proline residues 130 100.0%
Number of end-residues (excl. Gly and Pro) 2
Number of glycine residues (shown as triangles) 8
Number of proline residues 4
Total number of residues 144
Based on an analysis of 118 structures of resolution of at least 2.0 Angstroms
and R-factor no greater than 20%, a good quality model would be expected
to have over 90% in the most favoured regions.
2257088_01.ps

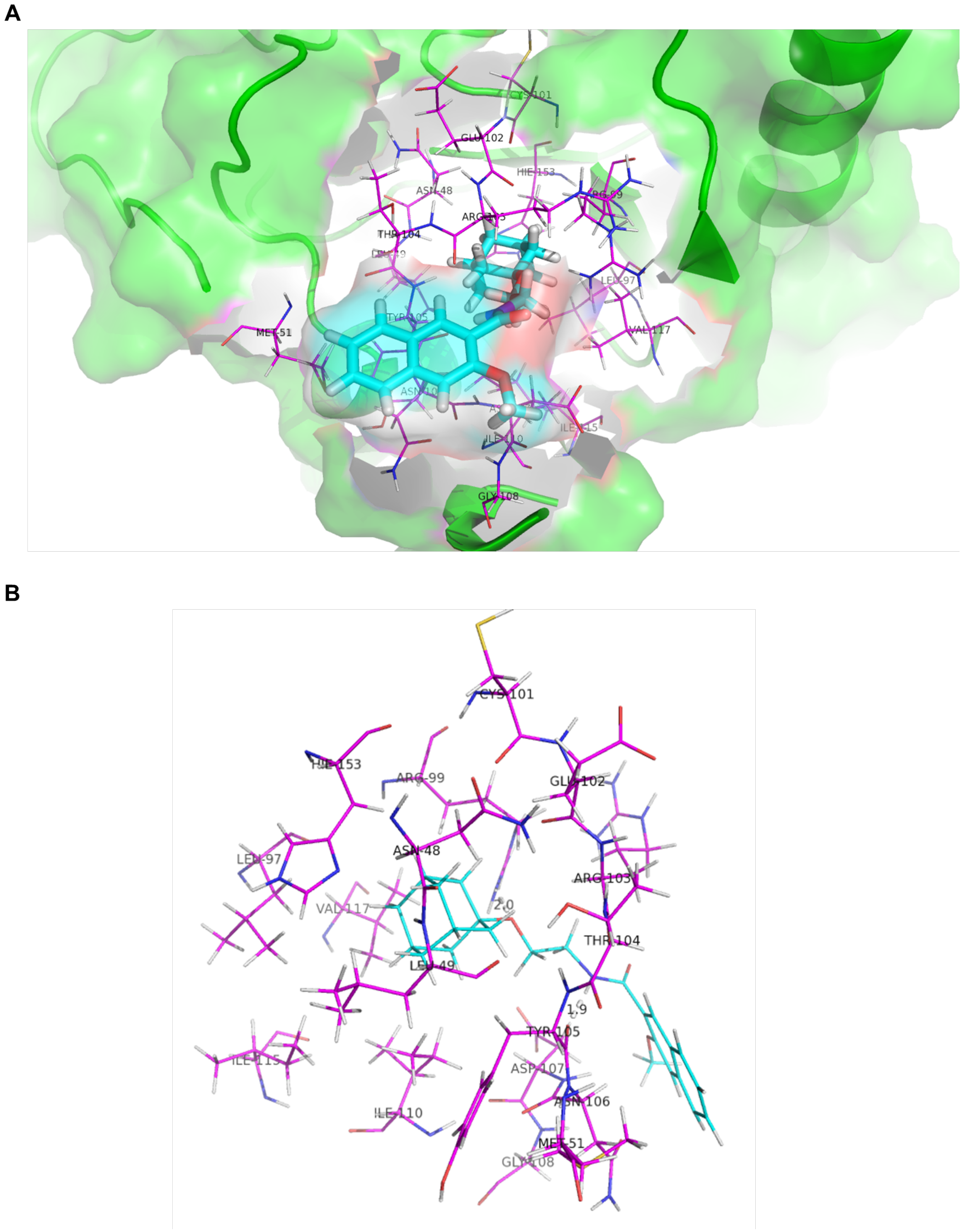
A
CYS 101
GLU 102
HIE 153
ASN 48
THR 104
TYR 105
MET 51
VAL 117
ILE 110
GLY 108
B
CYS 101
HIE 153
ARG 99
GLU 102
ASN 48
LEU 97
ARG 103
2.0
VAL 117
THR 104
LEU 49
1.9
TYR 105
ASP 107
ILE 115
ASN 106
ILE 110
MET 51
GLY 108

C

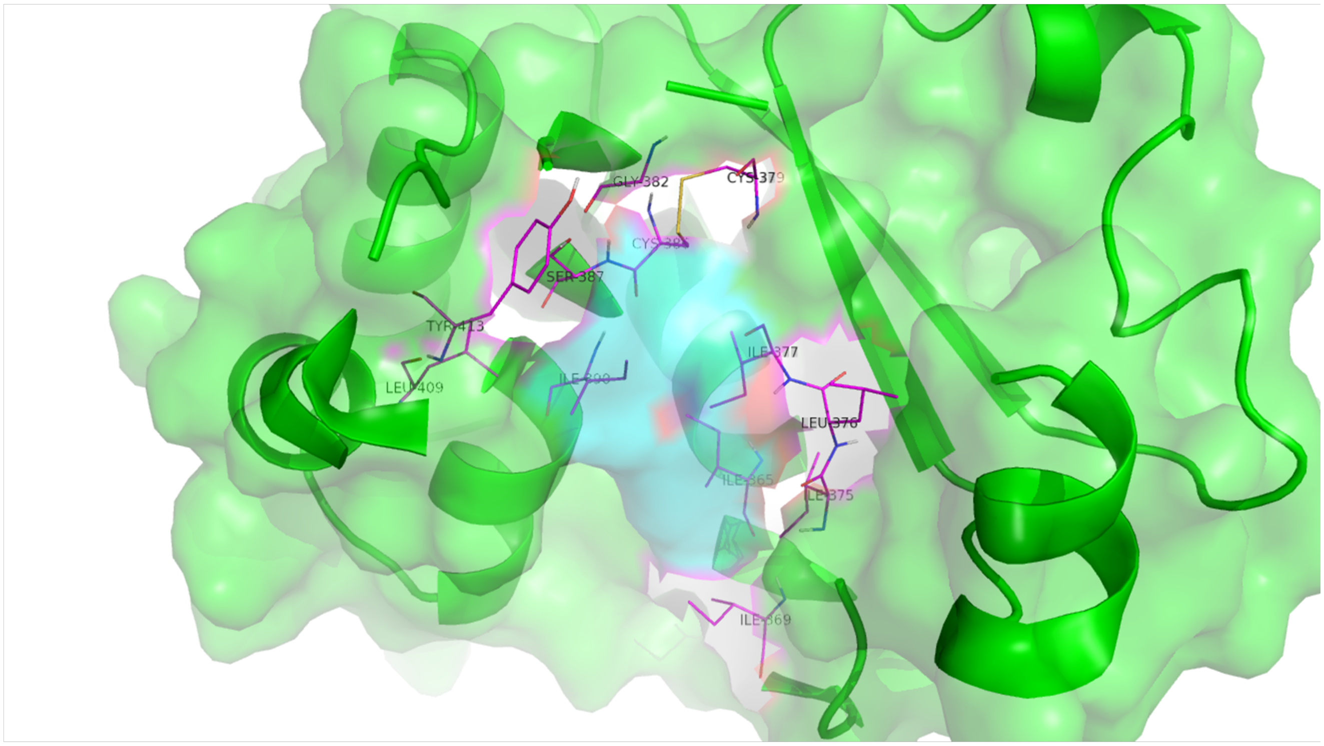
GLY 382
CYS 379
SER 387
TYR 413
LEU 409
ILE 377
LEU 376
ILE 365
ILE 375
ILE 369

D

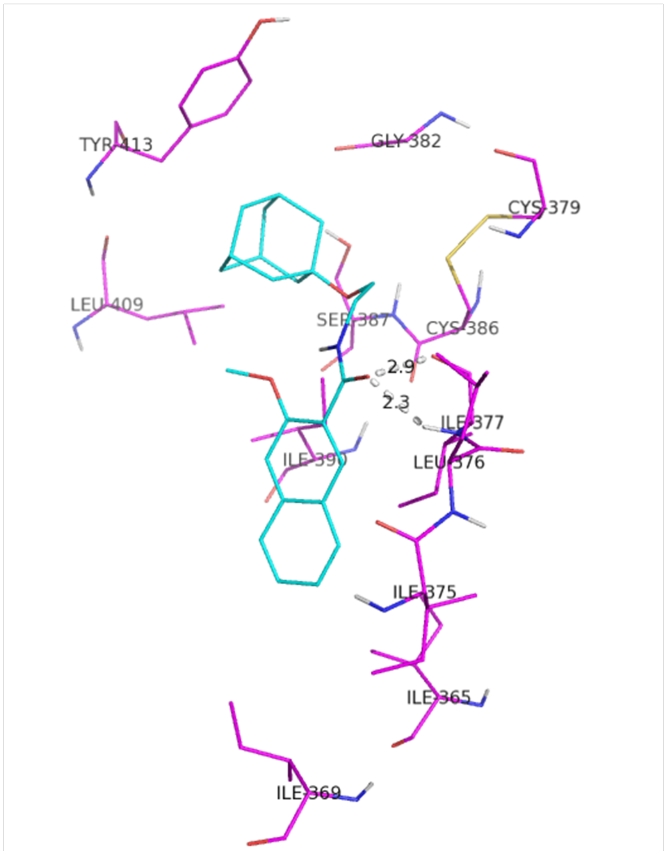
TYR 413
GLY 382
CYS 379
LEU 409
SER 387
CYS 386
2.9
2.3
ILE 377
LEU 376
ILE 375
ILE 365
ILE 369

E

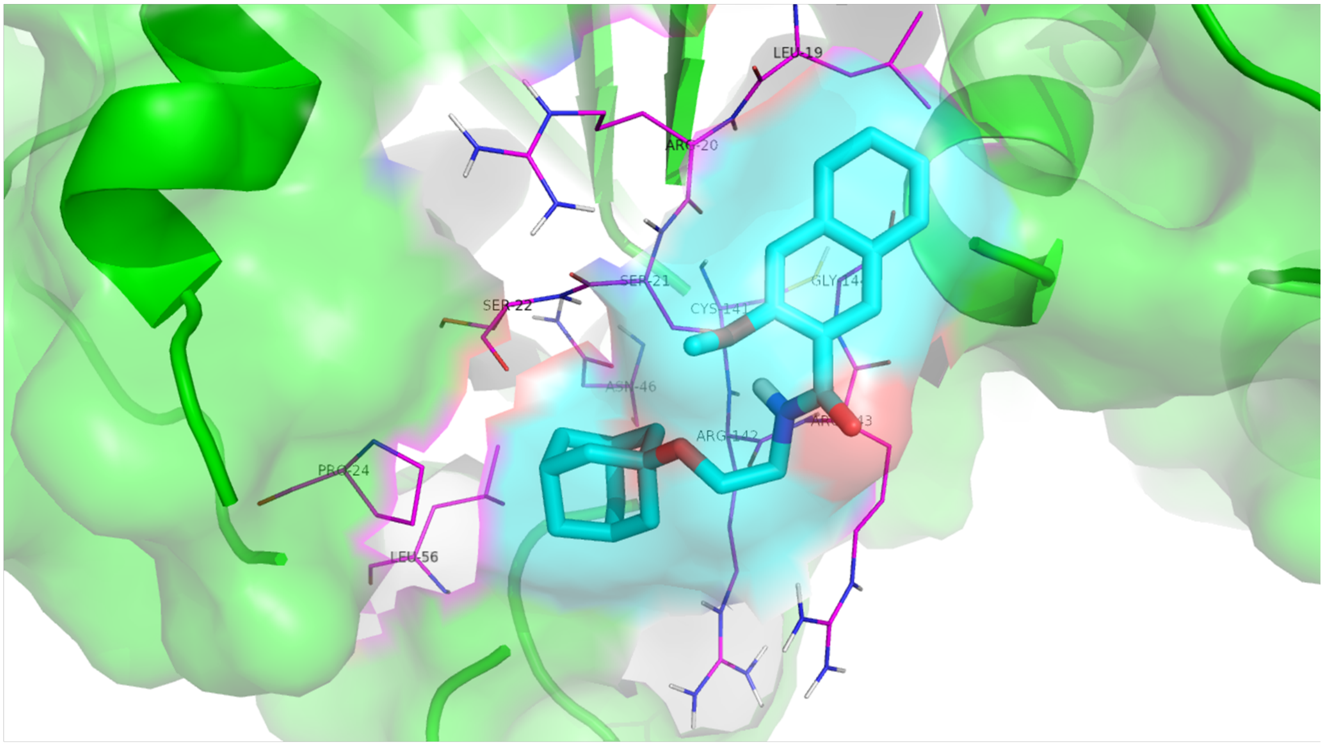
LEU-19
ARG-20
SER-21
SER-22
CYS-141
GLY-144
ASN-46
ARG-142
PRO-24
LEU-56

F

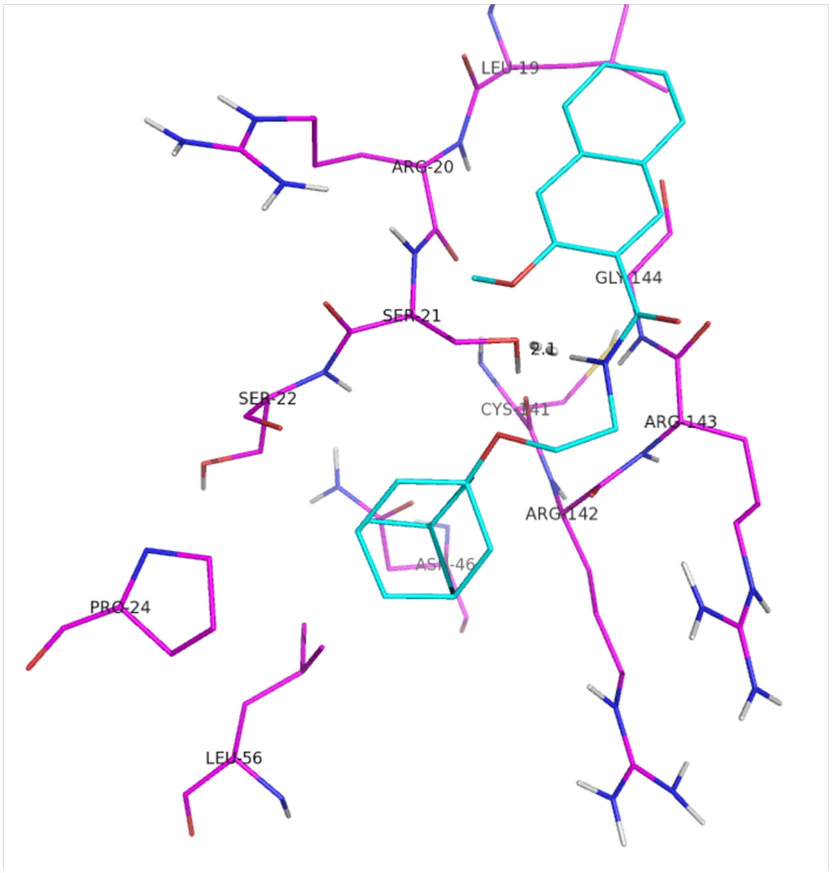
LEU-19
ARG-20
GLY-144
SER-21
2.1
SER-22
CYS-141
ARG-143
ARG-142
ASN-46
PRO-24
LEU-56

G

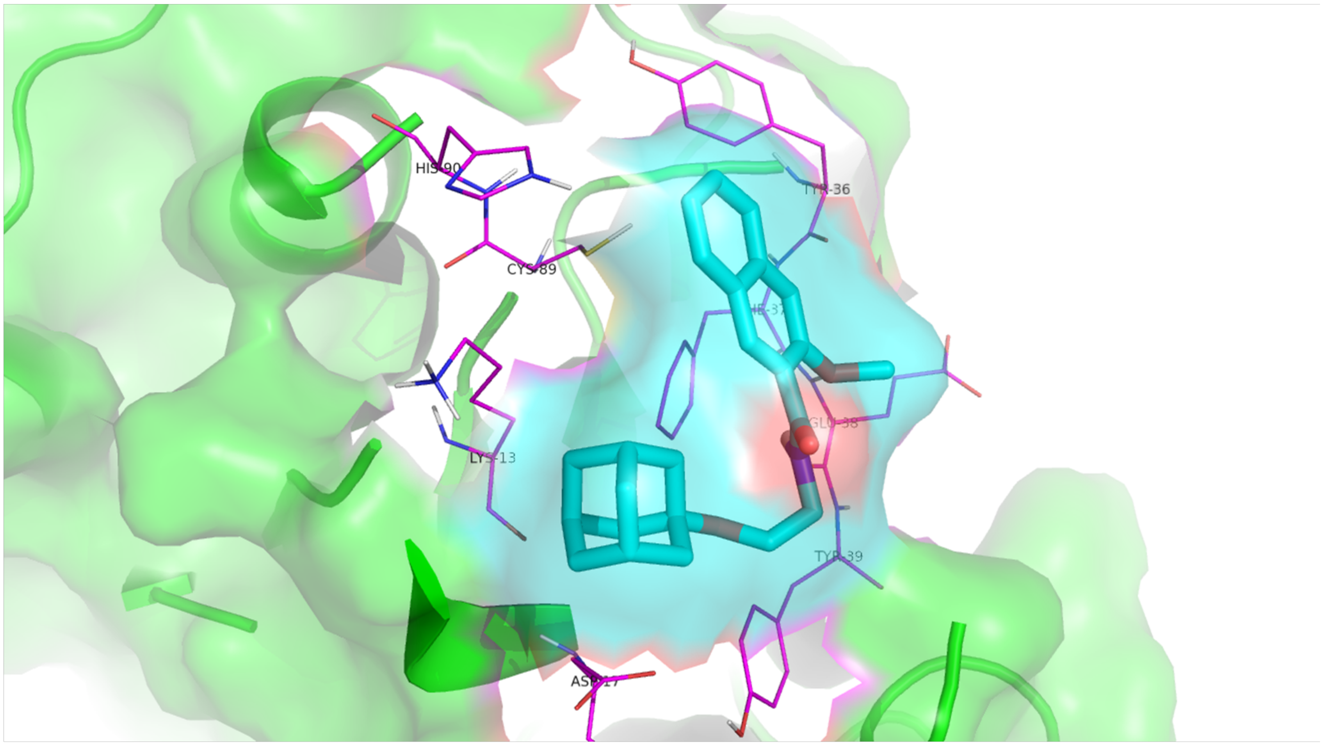
HIS-90
CYS-89
TYR-36
LYS-13
GLU-38
TYR-39
ASP-17

H

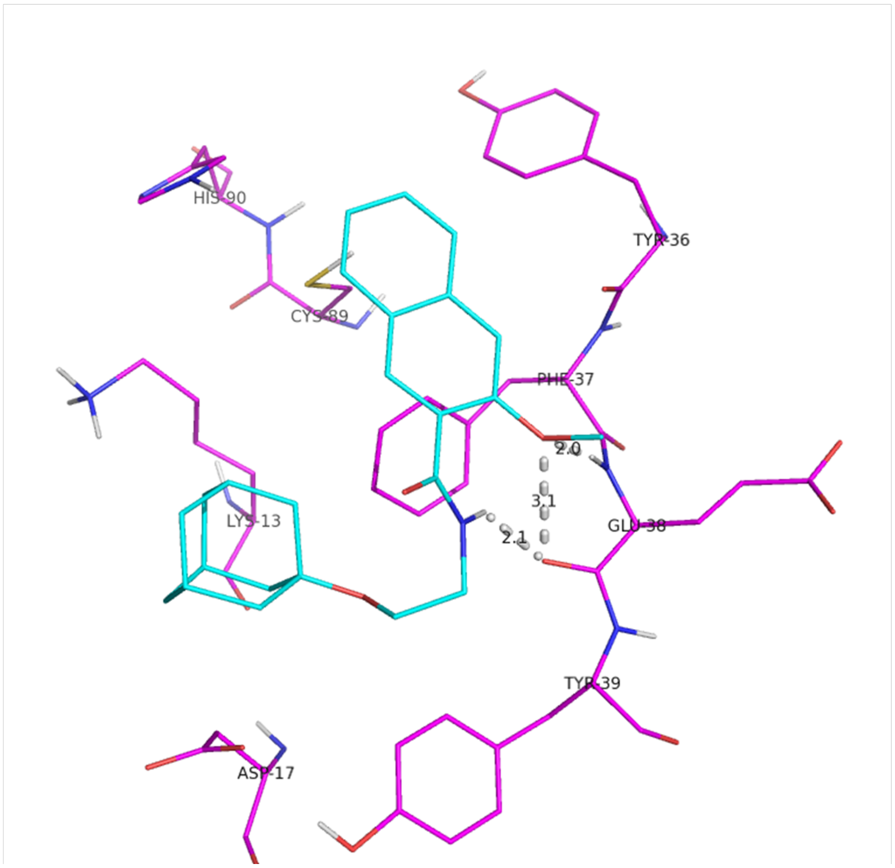
HIS-90
TYR-36
CYS-89
PHE-37
2.0
3.1
GLU-38
2.1
LYS-13
TYR-39
ASP-17

Figure 1A raw

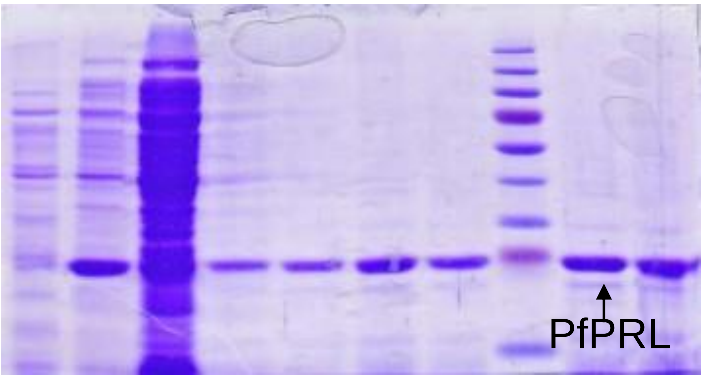

Figure 1B raw

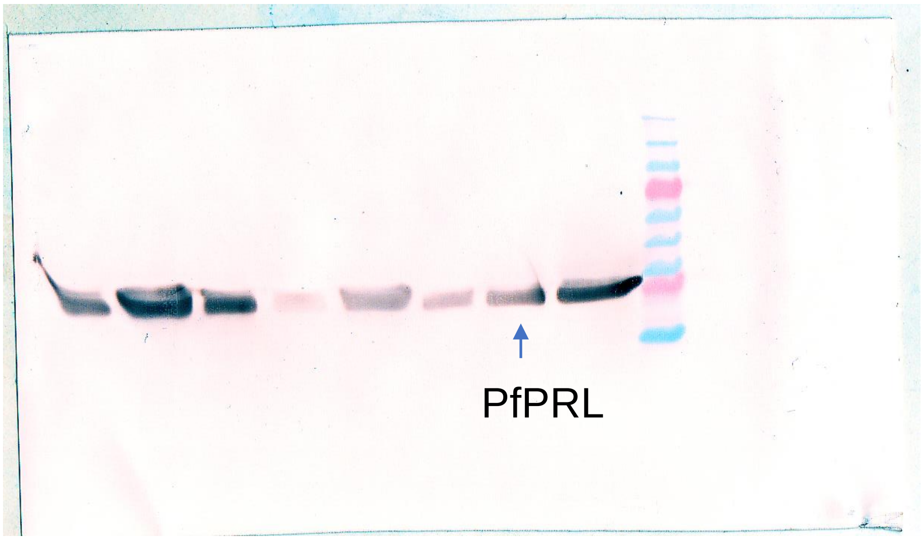

Supplement: Supplementary file 1 — Fig. S1. Recombinant PfPRL expression. (A) PCR amplified PfPRL using Q5 polymerase, (B) BamHI and SalI digested PfPRL clone in PJET, (C) Subcellular localization of recombinant PfPRL protein in E. coli expression system. Fig. S2. Multiple sequence alignment of four Plasmodium PTPs with conserved HCX5R motif. Fig. S3. Molecular dynamics simulation showing stable conformations of the predicted 3D structures of PF3D7_0309000. (A) Number of energy minimization steps required to achieve maximum force less than 1000 kJ mol‐1 nm‐1. (B) Fluctuations in temperature at constant volume (isothermal‐isochoric process) show that the system reaches the target temperature (300K) quickly and remain stable over the remainder of the equilibration. (C–D) Fluctuations in pressure and density at a constant temperature. (E–F) Root mean square deviation (RMSD) calculation using protein backbone structure and radius of gyration fluctuations during the 10 ns production simulation. Each analysis shows fluctuations within 2Å, suggesting correct and stable protein fold prediction for PF3D7_0309000. Additionally, during 10ns molecular dynamics run, the protein structure did not break, confirming stable predicted 3D structure. (G) Ramachandran plot analysis to check the quality of 3D model. Fig. S4. Molecular dynamics simulation showing stable conformations of the predicted 3D structures of PF3D7_1127000. (A) Number of energy minimization steps required to achieve maximum force less than 1000 kJ mol‐1 nm‐1. (B) Fluctuations in temperature at constant volume (isothermal‐isochoric process) show that the system reaches the target temperature (300K) quickly and remain stable over the remainder of the equilibration. (C–D) Fluctuations in pressure and density at a constant temperature. (E–F) Root mean square deviation (RMSD) calculation using protein backbone structure and radius of gyration fluctuations during the 10 ns production simulation. Each analysis shows fluctuations within 2Å, suggesting corr [file FEB4-11-1921-s001.pdf]
